# Supplementary material for: Molecular orbital breaking in photo-mediated organosilicon Schiff base ferroelectric crystals
Source: Nat Commun. 2024 May 24;15:4416. doi: 10.1038/s41467-024-48405-y (PMC11126662; doi:10.1038/s41467-024-48405-y)
Supplement: Supplementary file 1 — Supplementary Information [file 41467_2024_48405_MOESM1_ESM.pdf]

Supplementary Information

Molecular Orbital Breaking in Photo-Mediated Organosilicon Schiff base Ferroelectric Crystals

Gu *et al*.

Supplementary discussion

Significance:

Ferroelectric materials are widely used in various applications such as data storage, sensors, and transducers because of their robust spontaneous electrical polarization[1]. They generally undergo phase transitions which can be described as spatial symmetry breaking by Landau's phenomenological theory[2, 3]. Phase transition is essential for ferroelectrics since it can bring about tunable physical properties[4]. The phase transition mechanism of ferroelectrics mainly includes two types: order-disorder and displacive type. The former is usually found in some molecular ferroelectrics, while the latter is common in inorganic ceramics such as $BaTiO_3$ and $Pb(Ti, Zr)O_3$[5]. These processes are accompanied by changes in symmetric elements, which is well-known as symmetry breaking. Symmetry breaking will contribute to the rearrangement of ferroic orders and changes in spontaneous polarization, thereby achieving regulation of ferroelectric properties[6].

Recently, a new type of ferroelectric material, photo-mediated ferroelectric whose spontaneous polarization can be switched reversibly with a photoinduced phase transition triggered by structural photoisomerization has been proposed. This is a breakthrough in next-generation ferroelectric materials because photoirradiation stands out as a nondestructive, noncontact, and remote-control mean beyond an electric or strain field[7]. Among them, the typical photochromic compounds, such as Schiff base, azobenzene, diarylethene derivative, and spiropyran, have been chosen for light-driven ferroelectric materials, whose structural change is generally caused by photoisomerization instead of the traditional thermodynamic structural phase transition[8, 9]. However, the conventional phase transition mechanism cannot be used to describe them. Thus, a new ferroelectric phase transition driven by switchable covalent bonds is proposed. Molecular orbital breaking can help us to understand this unconventional ferroelectric mechanism from the perspective of electronics, and further provides a new approach to modulating ferroelectric properties at the electronic level especially the polarization, which is a beneficial supplement to

symmetry breaking. The core content of molecular orbital breaking is the valence bond recombination, for which the phase transition process involves the breaking and reformation of covalent bonds and thus leads to polarization changes. For example, Liao *et al.* disclosed the dual breaking of molecular orbitals and spatial symmetry in a photochromic diarylethene ferroelectric material[10]. This is the first time that this new ferroelectric mechanism–molecular orbital breaking is proposed. Meanwhile, such a photoinduced phase transition is entirely driven by switchable covalent bonds with breaking and reformation, enabling the reversible light-controllable ferroelectric polarization switching, dielectric, and nonlinear optical bistability. It should be highlighted that in comparison with traditional ones driven by thermal stimuli, photo-mediated ferroelectric materials will have promising application prospects in biomedicine since their physical properties can be mediated by light at a temperature matching the human body, making them promising candidates for applications in personal health management, sports detection, electronic skin, and other fields[11].

Here, we synthesized a pair of organosilicon Schiff base ferroelectric crystals, (*R*)- and (*S*)-*N*-(3,5-di-tert-butylbenzylidene)-1-((triphenylsilyl)oxy)ethanamine. They show optically controlled phase transition accompanying the molecular orbital breaking. The molecular orbital breaking is manifested as the breaking and reformation of covalent bonds during the phase transition process, that is, the conversion between C=N and C–O in the enol form and C–N and C=O in the keto form. As expected, these organosilicon Schiff base compounds show good biocompatibility and promotion of cell proliferation in biological cells confirmed by the tests of cell viability and adhesion. These intriguing features enable the multi-channel modulation in physical properties without the changing of temperature, thus making *R*- and *S*-SEA-Si a beneficial supplement to traditional ferroelectrics whose switchable properties are generally realized by thermodynamics. Therefore, this finding extends the ferroelectric mechanism for the optically-controlled ferroelectrics and highlights the important and unique applications of this type of materials in biomedicine. This will attract the attention of scientists from multiple

disciplines such as chemistry, physics, electronics, biomedicine, and materials science.

Highlights:

1. The discovery of a pair of organosilicon Schiff base ferroelectric crystals having photo-mediated bistability with multiple physical channels such as dielectric, second-harmonic generation, and ferroelectric polarization.
2. We first used molecular orbital breaking to interpret the phase transition mechanism from the perspective of electronics in the Schiff base ferroelectric system.
3. Photo-mediated organosilicon Schiff base ferroelectrics with good biocompatibility are essential for bioelectronics because they can realize the modulation of physical properties in a moderate environment compared to conventional thermodynamics ferroelectrics.

Supplementary Figures

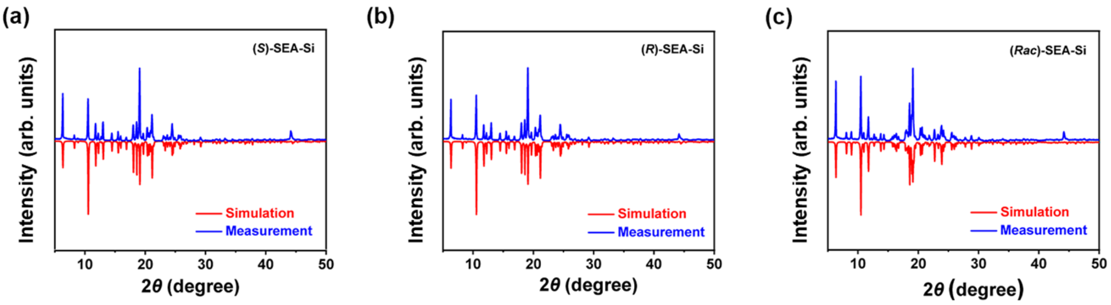

Supplementary Fig. 1 The comparison of the measured and simulated powder X-ray diffraction patterns of *S*-SEA-Si (a), *R*-SEA-Si (b), and *Rac*-SEA-Si (c).

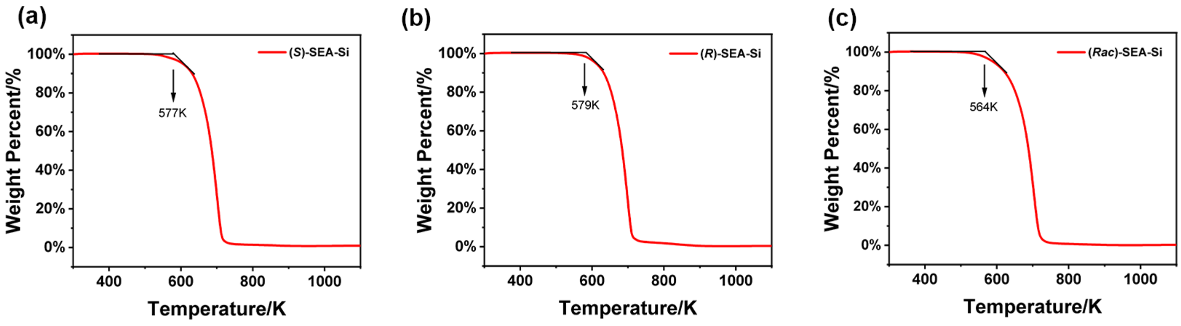

Supplementary Fig. 2 TGA curves of *S*-SEA-Si (a), *R*-SEA-Si (b) and *Rac*-SEA-Si (c).

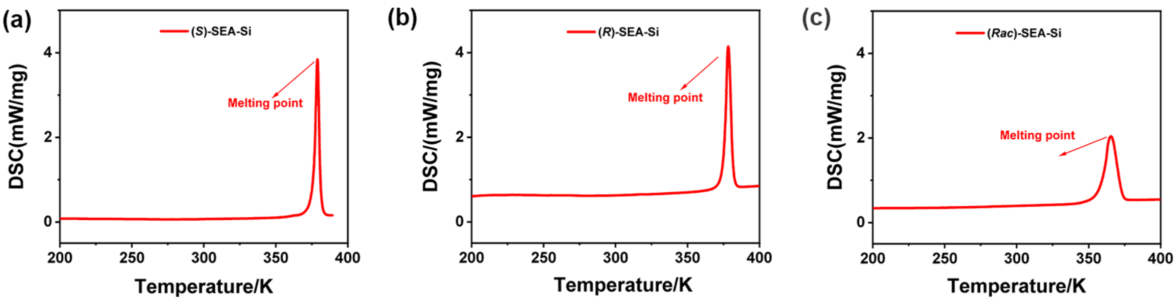

Supplementary Fig. 3 DSC curve of *S*-SEA-Si (a), *R*-SEA-Si (b) and *Rac*-SEA-Si (c).

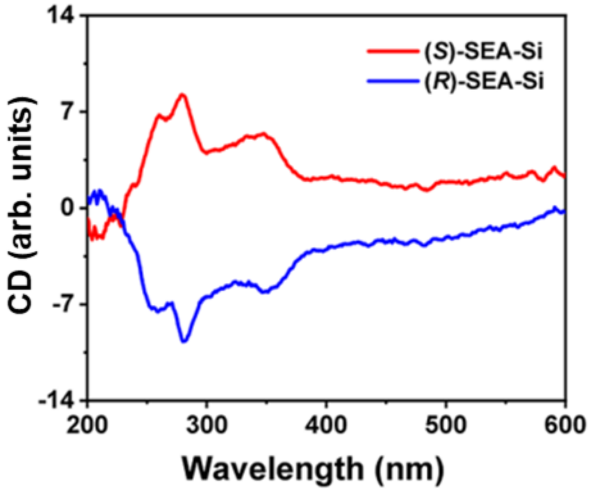

Supplementary Fig. 4 CD spectra of *S*-SEA-Si and *R*-SEA-Si.

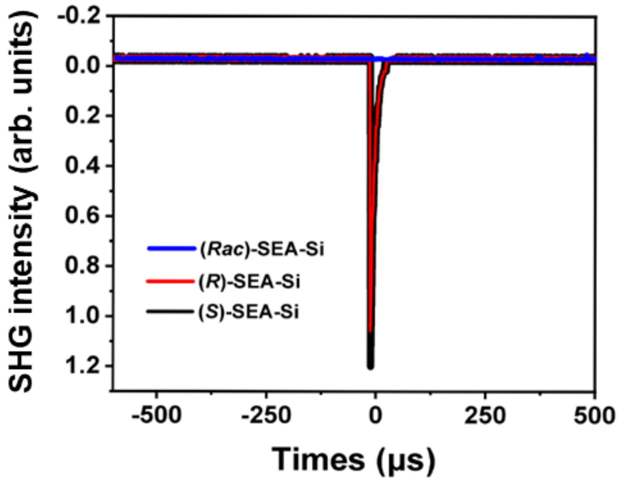

Supplementary Fig. 5 The SHG intensity of *S*-SEA-Si, *R*-SEA-Si, and *Rac*-SEA-Si.

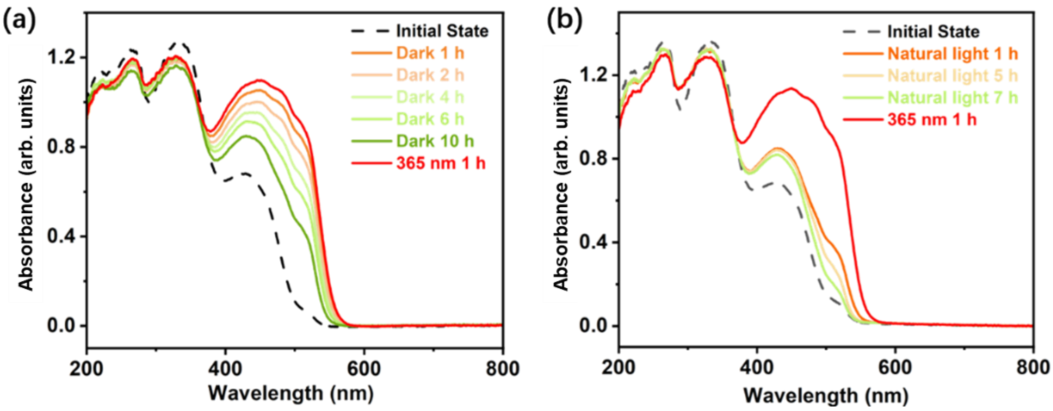

Supplementary Fig. 6 The time-dependent UV-vis spectra of *R*-SEA-Si irradiated with 365 nm for 1 hour: (a) in dark; (b) under natural light. The absorption intensity around 480 nm in *trans*-keto form gradually weakened and nearly dropped back within 1 h under natural light, suggesting that the recovery from *trans*-keto form to enol form in *R*-SEA-Si.

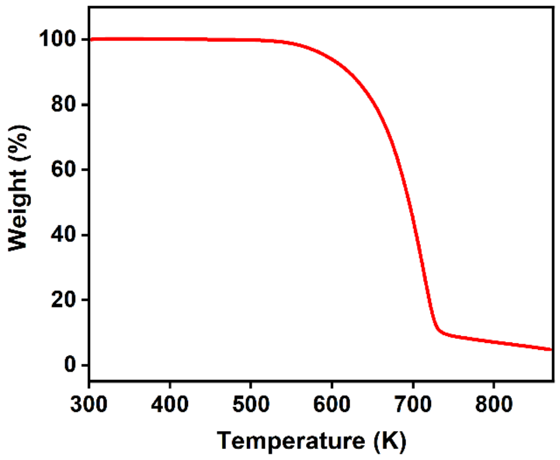

Supplementary Fig. 7 TGA curve of *R*-SEA-Si irradiated with 365 nm for 1 hour.

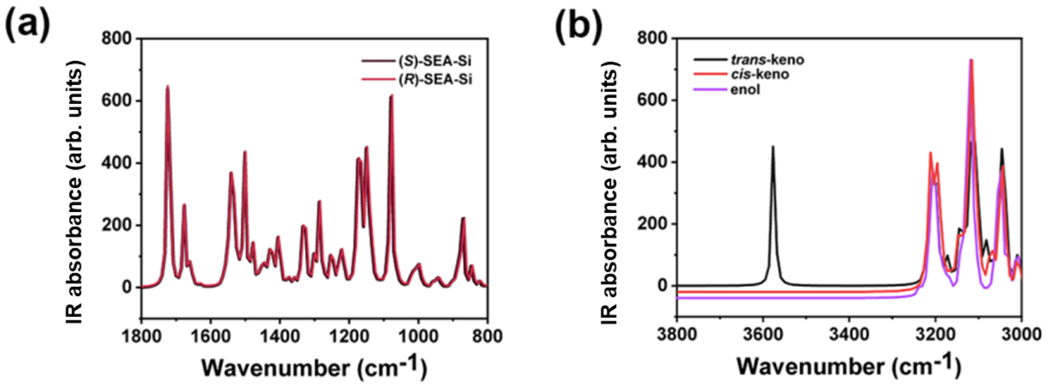

Supplementary Fig. 8 (a) Experimental IR absorption spectra of *S*-SEA-Si, *R*-SEA-Si at ambient conditions. (b) Calculated IR absorption spectra of *R*-SEA-Si with enol, cis-keto and trans-keto forms, respectively.

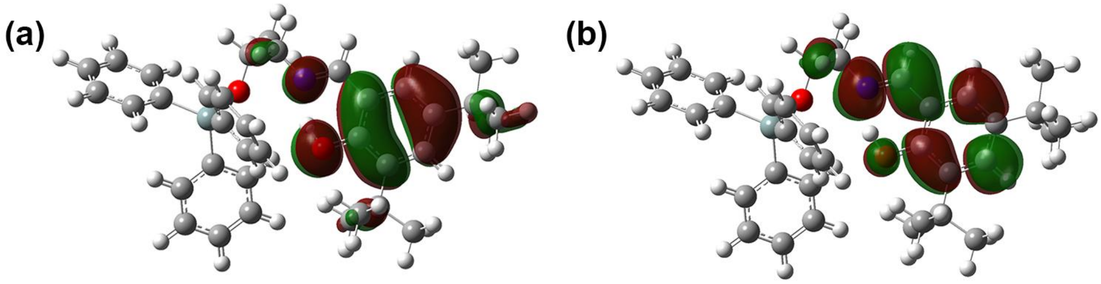

Supplementary Fig. 9 (a) HOMO and (b) LUMO of *R*-SEA-Si.

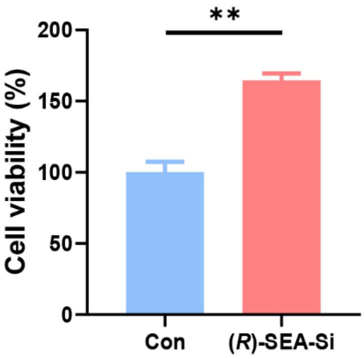

Supplementary Fig. 10 Cell viability of hBMSCs after incubation with the leach liquor of *R*-SEA-Si for 48 hours (n=6 per group).

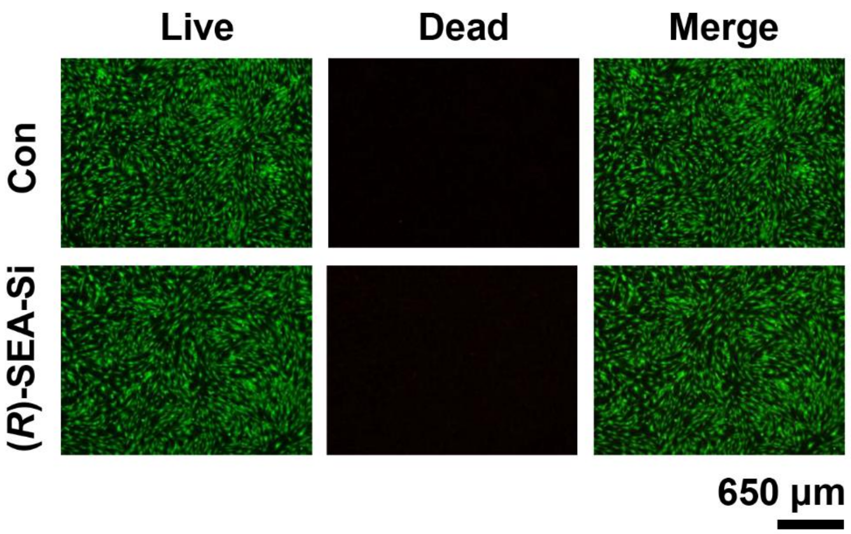

Supplementary Fig. 11 Representative scan of live/dead staining of hBMSCs after treated with leach liquor of *R*-SEA-Si for 48 hours, green and red labeled cells represent living and dead cells, respectively.

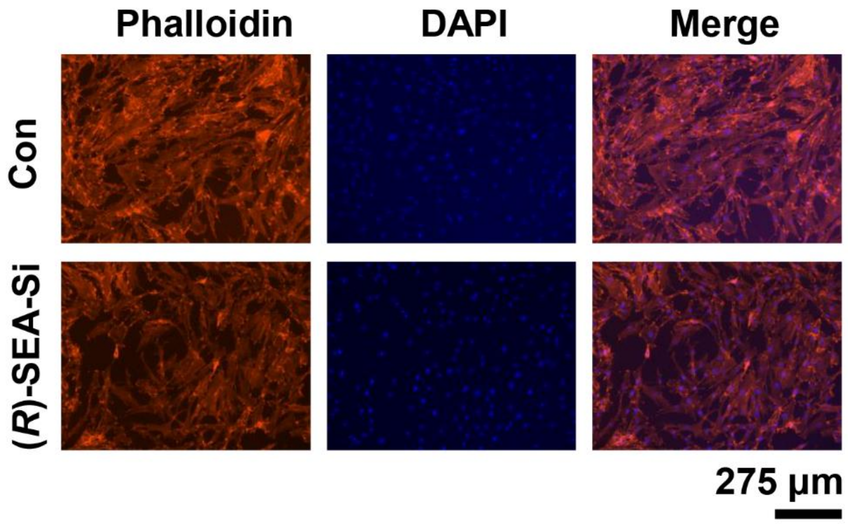

Supplementary Fig. 12 Representative scan of phalloidine/DAPI staining of hBMSCs after treated with leach liquor of *R*-SEA-Si for 48 hours, red and blue labeled cells represent cytoskeleton and nucleus of cells, respectively.

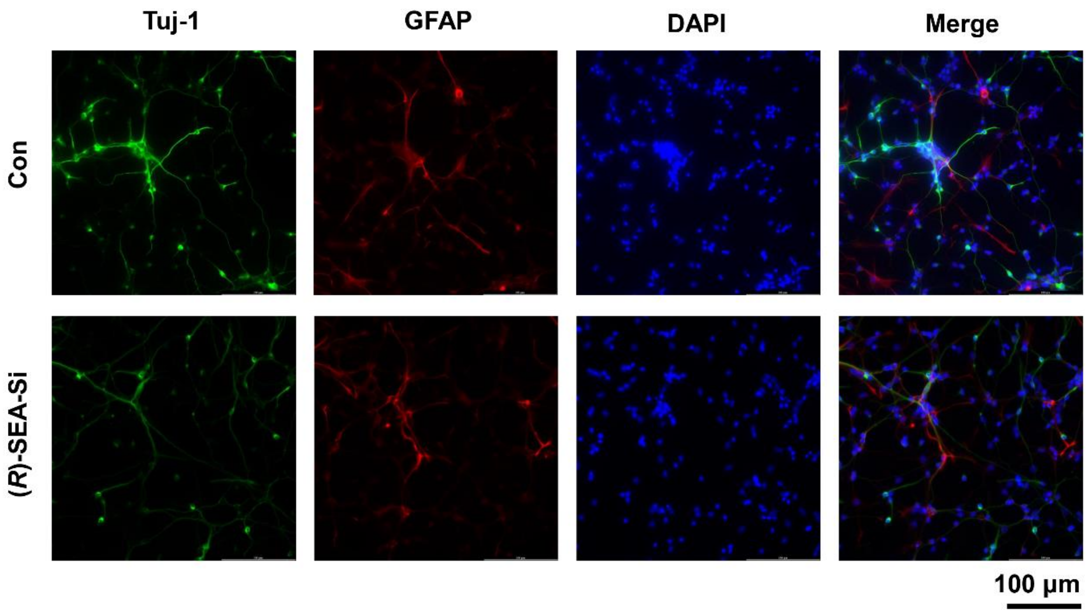

Supplementary Fig. 13 Immunostaining images against Tuj-1 (neurons, green), GFAP (astrocytes, red) and DAPI (nucleus, blue).

Supplementary Table

Supplementary Table 1: Crystal data and structure refinement for *S*-SEA-Si, *R*-SEA-Si, and *Rac*-SEA-Si.

Compound	*S*-SEA-Si	*R*-SEA-Si	*Rac*-SEA-Si
Empirical formula	$C_{36}H_{43}NO_2Si$	$C_{36}H_{43}NO_2Si$	$C_{36}H_{43}NO_2Si$
Formula weight	549.80	549.80	549.80
Crystal system	Monoclinic	Monoclinic	Monoclinic
Space group	$P2_1$	$P2_1$	$P2_1/c$
Temperature (K)	223 K	223 K	223 K
a (Å)	11.07925(8)	11.08297(11)	10.9470(1)
b (Å)	10.46988(7)	10.46765(9)	10.5650(1)
c (Å)	14.42836(12)	14.43065(15)	27.9640(2)
α (°)	90	90	90
β (°)	106.4077(8)	106.4332(11)	98.025(1)
γ (°)	90	90	90
Volume (Å^3)	1605.51(2)	1605.75(3)	3202.51(5)
Z	4	4	4
GOF	1.030	1.058	1.055
R_1 [$I > 2\sigma(I)$]	0.0284	0.0278	0.0394
wR_2 [$I > 2\sigma(I)$]	0.0792	0.0769	0.1093
Radiation type	Cu Kα	Cu Kα	Cu Kα

Supplementary References

1. You Y-M*, et al.* An organic-inorganic perovskite ferroelectric with large piezoelectric response. *Science* **357**, 306-309 (2017).

2. Rodriguez-Nieva JF, Scheurer MS. Identifying topological order through unsupervised machine learning. *Nat. Phys.* **15**, 790-795 (2019).

3. Cowley RA. Structural phase transitions I. Landau theory. *Adv. Phys.* **29**, 1-110 (1980).

4. Wesley Jeevadason A, Kalidasa Murugavel K, Neelakantan MA. Review on Schiff bases and their metal complexes as organic photovoltaic materials. *Renewable Sustainable Energy Rev.* **36**, 220-227 (2014).

5. Heng-Yun Ye Y-YT, Peng-Fei Li, Wei-Qiang Liao, Ji-Xing Gao,, Xiu-Ni Hua HC, Ping-Ping Shi, Yu-Meng You, Ren-Gen Xiong. Metal-free three-dimensional perovskite ferroelectrics. *Science* **361**, 151–155 (2018).

6. Shi P-P*, et al.* Symmetry breaking in molecular ferroelectrics. *Chem. Soc. Rev.* **45**, 3811–3827 (2016).

7. Tang YY*, et al.* Optical Control of Polarization Switching in a Single-Component Organic Ferroelectric Crystal. *J. Am. Chem. Soc.* **143**, 13816–13823 (2021).

8. Kobatake S, Takami S, Muto H, Ishikawa T, Irie M. Rapid and reversible shape changes of molecular crystals on photoirradiation. *Nature* **446**, 778-781 (2007).

9. Irie M, Fukaminato T, Matsuda K, Kobatake S. Photochromism of Diarylethene Molecules and Crystals: Memories, Switches, and Actuators. *Chem. Rev.* **114**, 12174-12277 (2014).

10. Liao WQ*, et al.* Dual Breaking of Molecular Orbitals and Spatial Symmetry in an Optically Controlled Ferroelectric. *Adv. Mater.* **35**, e2305471 (2023).

11. Zuo Y, Liang X, Yin J, Gou Z, Lin W. Understanding the significant role of SiOSi bonds: Organosilicon materials as powerful platforms for bioimaging. *Coord. Chem. Rev.* **447**, 214166 (2021).
