# Supplementary material for: Molecular orbital breaking in photo-mediated organosilicon Schiff base ferroelectric crystals
Source: Nat Commun. 2024 May 24;15:4416. doi: 10.1038/s41467-024-48405-y (PMC11126662; doi:10.1038/s41467-024-48405-y)
Supplement: Supplementary file 3 — Reporting Summary [file 41467_2024_48405_MOESM3_ESM.pdf]

nature portfolio

Corresponding author(s): Han-Yue Zhang
Last updated by author(s): Apr 20, 2024

Reporting Summary

Nature Portfolio wishes to improve the reproducibility of the work that we publish. This form provides structure for consistency and transparency in reporting. For further information on Nature Portfolio policies, see our Editorial Policies and the Editorial Policy Checklist.

Statistics

For all statistical analyses, confirm that the following items are present in the figure legend, table legend, main text, or Methods section.

n/a	Confirmed	
☐	☒	The exact sample size (*n*) for each experimental group/condition, given as a discrete number and unit of measurement
☐	☒	A statement on whether measurements were taken from distinct samples or whether the same sample was measured repeatedly
☐	☒	The statistical test(s) used AND whether they are one- or two-sided<br>*Only common tests should be described solely by name; describe more complex techniques in the Methods section.*
☒	☐	A description of all covariates tested
☒	☐	A description of any assumptions or corrections, such as tests of normality and adjustment for multiple comparisons
☐	☒	A full description of the statistical parameters including central tendency (e.g. means) or other basic estimates (e.g. regression coefficient) AND variation (e.g. standard deviation) or associated estimates of uncertainty (e.g. confidence intervals)
☒	☐	For null hypothesis testing, the test statistic (e.g. *F*, *t*, *r*) with confidence intervals, effect sizes, degrees of freedom and *P* value noted<br>*Give P values as exact values whenever suitable.*
☒	☐	For Bayesian analysis, information on the choice of priors and Markov chain Monte Carlo settings
☒	☐	For hierarchical and complex designs, identification of the appropriate level for tests and full reporting of outcomes
☒	☐	Estimates of effect sizes (e.g. Cohen's *d*, Pearson's *r*), indicating how they were calculated

Our web collection on statistics for biologists contains articles on many of the points above.

Software and code

Policy information about availability of computer code

Data collection	No software was used
Data analysis	No software was used

For manuscripts utilizing custom algorithms or software that are central to the research but not yet described in published literature, software must be made available to editors and reviewers. We strongly encourage code deposition in a community repository (e.g. GitHub). See the Nature Portfolio guidelines for submitting code & software for further information.

Data

Policy information about availability of data

All manuscripts must include a data availability statement. This statement should provide the following information, where applicable:

- Accession codes, unique identifiers, or web links for publicly available datasets
- A description of any restrictions on data availability
- For clinical datasets or third party data, please ensure that the statement adheres to our policy

All data are in a publicly accessible repository. The crystal structures generated in this study have been deposited in the Cambridge Crystallographic Data Centre under accession code CCDC: 2301143-2301145 and can be obtained free of charge from the CCDC via www.ccdc.cam.ac.uk/data_request/cif.

Research involving human participants, their data, or biological material

Policy information about studies with human participants or human data. See also policy information about sex, gender (identity/presentation), and sexual orientation and race, ethnicity and racism.

Reporting on sex and gender	This study does not involve this aspect.
Reporting on race, ethnicity, or other socially relevant groupings	This study does not involve this aspect.
Population characteristics	This study does not involve this aspect.
Recruitment	This study does not involve this aspect.
Ethics oversight	This study does not involve this aspect.

Note that full information on the approval of the study protocol must also be provided in the manuscript.

Field-specific reporting

Please select the one below that is the best fit for your research. If you are not sure, read the appropriate sections before making your selection.

☐ Life sciences ☐ Behavioural & social sciences ☒ Ecological, evolutionary & environmental sciences

For a reference copy of the document with all sections, see nature.com/documents/nr-reporting-summary-flat.pdf

Ecological, evolutionary & environmental sciences study design

All studies must disclose on these points even when the disclosure is negative.

Study description	We synthesize a pair of organosilicon Schiff base ferroelectric crystals, (R)- and (S)-N-(3,5-di-tert-butylbenzylidene)-1-((triphenylsilyl)oxy)ethanamine, which show optically controlled phase transition accompanying the molecular orbital breaking.
Research sample	(R)- and (S)-N-(3,5-di-tert-butylbenzylidene)-1-((triphenylsilyl)oxy)ethanamine crystals are square crystals with a particle size less than one millimeter.
Sampling strategy	The crystals of (R)- and (S)-N-(3,5-di-tert-butylbenzylidene)-1-((triphenylsilyl)oxy)ethanamine are obtained by slowly evaporating the methanol solution at room temperature.
Data collection	Z.-X.G., N.Z., and Y.Z. performed the physical characterization by using DSC, TGA, PFM, and other devices. Y.Z. and H.-Y.Z. carried out the analysis on computer. Z.-X.G., B.L., P.W., and Q.J. performed the in vitro biocompatibility and in vivo biosafety assay.
Timing and spatial scale	Data collection for this article began in March 2023 and ended in April 2024. The crystal sample is firstly synthesized, and then its physical properties were characterized, including phase transition, ferroelectric and optical properties. Each characterization experiment process involves many repetitions (June 2023-September 2023, January 2024-April 2024), so this step takes the longest time, is complex and has no specific sequence.
Data exclusions	No data were excluded from the analyses.
Reproducibility	All attempts to repeat the experiment were successful.
Randomization	The randomization is not relevant to our study, because the structure of the crystal samples obtained by repeated synthesis is determined, and the purity can reach 100%.
Blinding	We have arranged for two authors to conduct experiments independently, characterizing samples and collecting data at different times, to ensure that the final results are consistent.

Did the study involve field work? ☐ Yes ☒ No

Reporting for specific materials, systems and methods

We require information from authors about some types of materials, experimental systems and methods used in many studies. Here, indicate whether each material, system or method listed is relevant to your study. If you are not sure if a list item applies to your research, read the appropriate section before selecting a response.

Materials & experimental systems

n/a	Involved in the study
☒	☐ Antibodies
☐	☒ Eukaryotic cell lines
☒	☐ Palaeontology and archaeology
☐	☒ Animals and other organisms
☒	☐ Clinical data
☒	☐ Dual use research of concern
☒	☐ Plants

Methods

n/a	Involved in the study
☒	☐ ChIP-seq
☒	☐ Flow cytometry
☒	☐ MRI-based neuroimaging

Eukaryotic cell lines

Policy information about cell lines and Sex and Gender in Research

Cell line source(s)	Mouse calvarial pre-osteoblast cell line MC3T3-E1 cells and human bone marrow-derived mesenchymal stem cells (hBMSCs) were obtained from the Institute of Life Science Cell Culture Center (Shanghai, China)
Authentication	The MC3T3-E1 cells and hBMSCs were authenticated by the Institute of Life Science Cell Culture Center (Shanghai, China)
Mycoplasma contamination	The MC3T3-E1 cells and hBMSCs were not tested for mycoplasma contamination
Commonly misidentified lines (See ICLAC register)	Not relevant

Animals and other research organisms

Policy information about studies involving animals; ARRIVE guidelines recommended for reporting animal research, and Sex and Gender in Research

Laboratory animals	24 male ICR mice (8 weeks) were obtained from the Laboratory Animal Center of Nanjing Drum Tower Hospital, Affiliated Hospital of Medical School, Nanjing University (China). The ICR mice were housed in a standard environment with a temperature of 24–26°C, humidity of 70–75%, a 12–hour light–dark cycle, typical laboratory meals.
Wild animals	The study did not involve wild animals
Reporting on sex	male ICR mice was considered for study
Field-collected samples	The study did not involve samples collected from the field
Ethics oversight	All the experimental protocols in this study were approved by the Committee of Nanjing Drum Tower Hospital, Affiliated Hospital of Medical School, Nanjing University.

Note that full information on the approval of the study protocol must also be provided in the manuscript.

Plants

Seed stocks	This study does not involve this aspect.
Novel plant genotypes	This study does not involve this aspect.
Authentication	This study does not involve this aspect.
